# Are healthful behavior change policies ever unethical?

**DOI:** 10.1057/s41271-022-00372-8

**Published:** 2022-10-26

**Authors:** R. Scott Braithwaite

**Affiliations:** Division of Comparative Effectiveness and Decision Science, Department of Population Health, New York University Grossman School of Medicine, 227 East 30th Street Office 607, New York, NY 10016 USA

**Keywords:** Behavior change, Policy, Population health, Wellbeing, Justice

## Abstract

Public health experts often assume that any policy promoting healthful behavior change is inherently and self-evidently ethical. This assumption is incorrect. This Viewpoint describes why evaluating the ethics of a policy to promote healthful behavior change should require (1) valuing consequences for wellbeing proportionately to consequences for health, (2) valuing changes to the distributional equity of health and wellbeing together with their aggregate improvement, and (3) anticipating and surveilling for unintended consequences sufficiently important to offset benefits. I illustrate these three requirements through a hypothetical salt restriction policy, which is unethical if it evokes strong preferences that detract from wellbeing, disproportionately confers health benefits to those who are already healthy, or elicits unintended consequences that offset health benefits. I discuss why analogies of salt restriction mandates are inappropriate. In summary, public health decision-makers should employ more structured, explicit and comprehensive criteria when considering the ethical consequences of policies.

## Key messages

- Health policymakers should consider the effects of health policies on overall wellbeing, not just on health.
- Health policymakers should try to anticipate how behavior change policies may lead to undesired and compensatory behavior changes.
- Although many health policies unintentionally worsen health inequities, methods exist to avoid this consequence.

## Introduction

Health improvement is clearly preferable to no health improvement. So why even bother to ask if a public health policy promoting healthful behavior change is unethical? First, behavior change has consequences for wellbeing (see Glossary) in addition to consequences for health. Second, beneficial consequences may accrue disproportionately to those who are already healthy, exacerbating health inequities. Third, policies may invoke unintended consequences that offset benefits, and which are likely to go unnoticed without appropriate forethought and surveillance by policymakers.

While these considerations are not new, recent scholarship is placing them into sharper focus by evolving notions of wellbeing and its relationship with health, and by elevating the importance of health equity. For example, a policy to restrict salt may improve health for salt-lovers, but may lower wellbeing for salt-lovers through effects observed in other dietary interventions, such as an enduring and offsetting decrement in gustatory satisfaction, a devaluation of the social or cultural context of consuming particular foods, or a stigmatization of their preferences [1]. A salt restriction policy may benefit those likely to stay healthy more than those likely to get sick, and may exacerbate health inequities [2]. It may lead salt-lovers to redirect their gustatory reward-seeking towards other unhealthy targets, such as sweet foods or calorically dense foods [1].

The example illustrates why ethics of public health decision-making is complex beyond the ethics of individual health decision-making. In public health decision-making as well as in individual health decision-making, domains of ethical valuation encompass autonomy and liberty, beneficence and non-malfeasance, distributive justice, and procedural justice (Glossary) [3–7]. In both cases, these domains may involve non-health as well as health consequences. In public health decision-making, the policy intervention may be more distinct from its health objective than a medical intervention is from its biological objective, leading to a wider scope of opportunities for unintended consequences. Additionally, in public health these domains are applied to populations rather than to particular individuals. Not only may the ethical calculus of a particular decision vary for different individuals, but the most appropriate distributive justice framework for applying this calculus to society has long been debated, along with accompanying tradeoffs between individual liberty and collectivism (Glossary) [8].

Glossary

- Wellbeing: Summary measure of quality-of-life, with subjective domains including affective (e.g., happiness), sensory (e.g. pleasure), and cognitive (e.g. satisfaction). Health is an important contributor to wellbeing but not the sole contributor. Related to the notion of “welfare” in economics
- Individual liberty: Elevates individual over society. Implies that individuals have the power and scope to act as they please (for example, to pursue wellbeing)
- Collectivism: Elevates society over individual. Implies that societal wellbeing transcends and is not deducible from the distribution or total amount of wellbeing of its individual members
- Beneficence: The principle of benefitting others
- Non-malfeasance: The principle of avoiding harm to others
- Procedural justice: The fairness of the process by which determinants of wellbeing (for example, health, wealth) are distributed in a society
- Distributive justice: The fairness of the distribution of determinants of wellbeing in a society, often judged based on the following principles:
  - Utilitarianism: Increasing a society’s total amount of wellbeing is vastly more important than equalizing its distribution
  - Egalitarianism: Equalizing a society’s distribution of wellbeing is vastly more important than increasing its total amount
  - Maximinism: The amount of wellbeing a society distributes to its worst-off is vastly more important than its total amount or other aspects of its distribution
  - Prioritarianism: Intermediate to utilitarianism and egalitarianism: gains to worse-off are more important than to gains to better-off to an extent that can be variably specified
  - Sufficientism: Amalgam of prioritarianism and utilitarianism; gains to worse-off are only more important than gains to better-off if a specifiable level of basic needs are not met  for the worse-off (or sufficiency)
- Ego depletion: The sensation of fatigue from sustained attention to a difficult task
- Mesolimbic reward circuitry: Neurological pathways that produce appetitive and/or aversive sensations, often to reward behaviors crucial for survival or to discourage behaviors that threaten survival

These concepts have arisen from extensive debate within and across multiple disciplines including economics, ethics, decision science, and the health sciences. Many are still evolving. This glossary dramatically simplifies and contextualizes them for this Viewpoint. Helpful references include the following:

1. Cooke, P.J., T.P. Melchert, and K. Connor, *Measuring Well-Being:A Review of Instruments.* The Counseling Psychologist, 2016. 44(5): p. 730–757.
2. Linton, M.-J., P. Dieppe, and A. Medina Lara, *Review of 99 self-report measures for assessing well-being in adults: Exploring dimensions of well-being and developments over time.* BMJ Open, 2016. 6: p. e010641.
3. Diener, E., S. Oishi, and L. Tay, *Advances in subjective well-being research.* Nat Hum Behav, 2018. 2(4): p. 253–260.
4. Zalta, E. N., Nodelman, U. Stanford Encyclopedia of Philosophy, 2022. https://plato.stanford.edu/

## Valuing consequences for wellbeing

Presuming that a healthful behavior change policy is ethical assumes that its effects on health have a favorable calculus of proportionality relative to its effects on wellbeing. Scholars have noted that public health decision-makers should strive for proportionality, balancing health objectives for improving population health and health equity against other compelling interests in the non-health sphere that may compromise wellbeing, such as liberty, privacy, avoiding social harms such as stigmatization, and avoiding undue financial burdens or opportunity costs [5]. This balance does not imply that all interests in the non-health sphere are sufficiently important to merit inclusion in the decision calculus. In particular, scholars have noted that asking whether liberties and other non-health interests are “consequential” rather than “routine,” or even more stringently, “central to determination,” can serve as a guide for assessing whether they merit consideration in the calculus of public health decisions [9].

Following from these principles, a policy is ethically undesirable for a particular individual if she would prefer to opt-out because she values the expected benefit to her health less than the expected detriment to her wellbeing in non-health domains. For example, a low salt diet would impair wellbeing for a salt-lover, who may be willing to tradeoff the health benefit from salt restriction in exchange for the increased wellbeing that comes from unrestricted salt access. More specifically, an adult at typical risk for cardiovascular disease and without known hypertension would gain approximately 2 weeks of life expectancy through the 1–2 mm average reduction in systolic blood pressure from lowering salt intake [10]. Accordingly, if that person would be willing to give up 2 weeks of life to live without salt restriction, then a low salt diet would have an unfavorable health-wellbeing calculus for that person [10]. Notably, persons with or at high risk for cardiovascular disease, or who have pre-hypertension, would have greater health gains from salt restriction, so any affinity for salt would be less likely to confer an offsetting decrement in wellbeing. On the other hand, persons at lower risk for cardiovascular disease would have lesser health gains, so any affinity for salt would be more likely to confer an offsetting decrement in wellbeing.

## Valuing consequences for equity

Presuming that a policy promoting healthful behavior change is ethical assumes that the population distribution of improvements in health is ethically desirable, whether judged from an egalitarian perspective, a utilitarian perspective, or any other normative perspective regarding distributive justice such as, prioritarianism, maximinism, or sufficientism (Glossary) [11]. If salt restriction offers less benefit for lower socioeconomic groups than for higher socioeconomic groups, as could happen if lower income people seek substitutes that are cheaper and therefore more likely to rely on fat and sugar for their palatability (such as calorically dense packaged foods) than the substitutes sought by higher income persons (such as herb-infused olive oils), the policy would augment health inequity between these groups. The ethical harm from augmenting health inequities could offset the ethical benefit from improving aggregate health.

Fortunately, there are established approaches to evaluate the ethical calculus of such trade-offs. In particular, public health practitioners can choose outcome metrics that adjust for inequality-aversion by simultaneously considering the ethical valence of changes in the distribution of health that may occur together with health improvement.

## Inequality-aversion

Inequality-aversion is a quantitative construct that reflects whether and by how much a society is willing to trade-off some aggregate benefit to achieve a more equal distribution of that benefit [12, 13]. The notion of inequality-aversion is rooted in economics, a discipline of scholars who have long questioned whether and how societal welfare (often proxied by distribution of per-capita income) is distinct from the aggregation of the welfare of individuals in that society (often proxied by per capita income alone). Health science researchers are increasingly adopting inequality-aversion because they increasingly recognize the importance of the analogous health question: to what extent is societal health (often proxied by distribution of healthy life years) distinct from the aggregation of the health of individuals in that society (often proxied by per-capita healthy life years alone).

Scholars commonly credit health-related specifications of inequality-aversion to Atkinson (1970) [12]. While he was one of many economists who devised formulae to quantify inequality, he was one of few who took the idea further by formulating an “inequality aversion parameter” (IEP) that quantifies a society’s strength of preference to trade-off some aggregate benefit to obtain a more equal distribution of that benefit. The IEP therefore specifies a degree of prioritarianism on the spectrum between egalitarianism and utilitarianism (Glossary). A society with little or no inequality-aversion would have an IEP close to 0, specifying minimal prioritarianism that converges with utilitarianism. In contrast, a society with vast inequality-aversion would have an IEP approaching infinity, specifying maximal prioritarianism that converges with egalitarianism.

To illustrate empirically grounded examples between these two extremes, if society members would prefer a scenario where everyone lives 60.0 healthy years to a scenario where half live 65.5 healthy years and half live 55.5 healthy years, accepting a loss of 0.5 healthy years in aggregate to attain an equal distribution, that society has moderate inequality-aversion (corresponding to an IEP of 3, approximating the midrange of empirically derived measurements of health inequality-aversion). If members of a society would prefer a scenario where everyone lives 60 healthy years to a scenario where half live 57.0 healthy years and half live 67.0 healthy years, accepting a loss of 2.0 healthy years to attain an equal distribution, the society has high inequality-aversion (corresponding to an IEP of approximately 10, towards the higher end of empirical measurements of health inequality-aversion). It is possible to elicit inequality-aversion with or without respect to a specified characteristic that co-varies with health and may compound the ethical valence of its unequal distribution. For example, in empirical studies, people in the United Kingdom (UK) display greater inequality-aversion if the health inequality compounds other manifestations of disadvantage such as socioeconomic status [12, 13].

Returning to the hypothetical salt restriction policy, if this policy added 2 weeks to a society’s average lifespan but this benefit was unequally distributed, with 3 weeks of additional life expectancy accruing to the healthiest sub-population (for example, life expectancy of 90 years) but only 1 week of additional life expectancy accruing to a similarly sized, unhealthiest sub-population (with life expectancy of 60 years), a societal inequality-aversion corresponding to a IEP of 10 would render this policy ethically equivalent to increasing the population life expectancy by only 1.03 weeks rather than 2 weeks, effectively cutting the benefit in half. Consequently, if alternative policies with similar societal costs could deliver an equally distributed benefit greater than 1.03 additional weeks of life expectancy and less than 2 additional weeks of life expectancy, the salt restriction policy could displace those other more highly valued uses of societal resources, and the salt restriction policy would become unethical. In this scenario, the salt restriction policy would appear ethical without considering distributional consequences but could become unethical when also considering distributional consequences.

## Anticipating and surveilling for indirect health consequences of public health policies

While health authorities design public health policies in general, and healthful behavior change policies in particular, to advance a particular health goal, these policies may have the unintended consequences of offsetting other health goals. But policymakers can sometimes anticipate unintended consequences, and can often conduct surveillance for them. Indeed, scholars have noted that regulators would not allow a blockbuster drug into the health market without extensive experimental evaluation to demonstrate efficacy and safety, but standards for policy are not as stringent [14].

## Anticipating

First, health-related goods and services, like other goods and services, often have complements (for example, goods or services that are consumed together) or substitutes (for example, goods or services consumed in place of another) [15]. A salt-lover who has reduced access to salt may substitute other highly palatable and rewarding foods such as those with high caloric density or sweetness. A salt-lover who only will eat fresh vegetables with the complement of added salt may elect to eat more packaged food. A policy restricting salt content in packaged foods may lead the makers of packaged foods to find other inexpensive ways of making food appealing, such as increasing their calorie density. Indeed, if low socioeconomic status persons cannot afford or access healthful substitutes, the disadvantage from low socioeconomic status might be compounded by disadvantage from poorer health.

Second, many individuals are subject to ego depletion (for example, self-regulation fatigue), which limits the capacity to sustain concentrated effort regardless of domain [16]. The phenomenon of ego-depletion explains why people who exert mental effort have less capability for subsequent physical effort and vice versa. Accordingly, people who must exert great effort to maintain low salt intake may be less capable of exerting effort towards other health goals such as exercising or restricting calorie intake. Unintended health consequences that are anticipatable should be included in the health calculus of a policy, and any health policy with a negative health calculus is likely to have a negative ethical calculus.

Considering non-health consequences is aided by measuring preferences. This seems dauntingly complex but is not. Before scaling a policy, policymakers could facilitate research to identify the most important and relevant preferences in the target population using qualitative methods such as focus groups and key informant interviews, and the strength of these preferences can be measured using a quantitative procedure such as discrete choice experiments or best–worst scaling [17]. This process may be viewed as analogous to identifying and valuing “preference-sensitive decisions” in health care, where it is especially important to elicit and include preferences in healthcare decision-making [18].

## Surveilling

Surveilling for undesired health consequences can be facilitated by piloting a new policy for a specified time or a specified sub-jurisdiction, or both, and gathering relevant data during that pilot. For example, suppose that health authorities are considering a salt restriction policy that would require makers of all prepared foods to limit their salt content to no more than a pre-specified quantity per serving.

A pilot of this program would enable monitoring for whether the health effects of salt reduction are countered by an increase in other unhealthy choices such as consuming more sugar or fat. A pilot would also enable monitoring for whether health inequity is worsened because people who are unhealthy (such as people with multiple social risks for poor health) are less likely to reduce their salt intake, or because lower socioeconomic status persons are more likely to make unhealthy substitutions. Piloting the salt restriction policy could also permit re-measurement of preferences to assess if these changed once the policy became real rather than hypothetical. If there is no signal of undesirable consequences during the pilot stage, than the policy can be scaled. Indeed, piloting policies prior to scaling would facilitate gathering evidence of long-term efficacy of a policy prior to making behavior change recommendations at the population level, and would provide opportunities for attaining established public health practice goals such as assessing responsiveness, reaffirming public consent, and for enhancing public participation and transparency [3].

## Why restricting salt does not have the same ethical calculus as mandating seatbelts

Anecdotal evidence suggests that many public health practitioners view seat belt requirements as reflecting a similar ethical calculus as salt restriction. However, it is difficult to overstate the enormity of qualitative and quantitative differences invoked by this comparison. Seat belts reduce motor vehicle fatalities by between 45 and 73%, and the lifetime chance of dying in a motor vehicle accident in the USA is 0.92% with current levels of seat belt use (86–88%) [19]. Therefore, without seatbelt use, the lifetime chance of dying in a motor vehicle accident would rise to approximately 2%, eliminating more than 1 year of life expectancy, more than the effect of all cancer screenings combined. Further, seat belts do not necessarily conflict with deep-seated cultural and behavioral norms or activators of the brain’s mesolimbic reward circuitry (Glossary) [20], a potent reinforcer of activities necessary for evolutionary success. The mesolimbic reward system reinforces activities that enhance survival and reproductive success in evolutionarily normative contexts of scarcity, uncertainty, and danger, including eating calorically dense food and obtaining salt and other necessary minerals. In a resource-rich society, these activities are no longer as necessary, but are still powerfully reinforced by the mesolimbic reward system. Very few people habituated to seatbelt use would prefer the freedom of seatbelt-free driving in exchange for losing more than a year of life. In contrast, a low salt diet adds only 2 weeks of life. Many salt-lovers habituated to impaired salt access may elect to return to unrestricted salt intake and the attendant mesolimbic reward in exchange for foregoing 2 weeks of life.

A presumption that health rarely conflicts with wellbeing is the norm in the public health community. However, while health and wellbeing are strongly correlated, exogenous actions to change health do not necessarily change wellbeing in the same direction, a distinction of great importance. Indeed, a blind spot regarding effects of public health policy on wellbeing was evident in the seminal writings of Geoffrey Rose, who advocated treating “sick populations” rather than “sick individuals” because a greater population health benefit can arise from unselectively lowering risk by a small amount for everyone in a population, than from selectively reducing risk by a large amount for the highest-risk individuals. Rose considered the idea that policies involving behavior change could have an unfavorable wellbeing calculus for low risk individuals, who have little to gain but just as much to lose from the disutility of behavior change. However, he was untroubled by the ethical implications of this wellbeing calculus because he thought that any disutility of behavior change would soon fade. Rose errantly presumed that disutility of behavior change arose from deviations from behavioral norms, which are malleable [21], rather than from mesolimbically reinforced and evolutionarily honed traits, which resist change.

## Limitations

Many public health ethicists will disagree with the reasoning outlined here. For example, some have argued that beneficence, non-malfeasance, and autonomy are less important in public health ethics than in other bioethical domains [22]. Others have argued that the ethics of public health is inseparable from the ethics surrounding contextual environmental factors linked to health, such as environmental protection [23], or to the social relationships that enable society to be sufficiently stable, resourced, and organized to pursue health.

These arguments have important additional limitations. First, they assume that any changes in normative standards induced by a policy would not sufficiently alter the benefit-to-harm calculus such that the policy would become favorable for a majority of persons. For example, if a salt restriction policy allowed a majority of salt-lovers to willingly forgo salt because they would be released from the normative pressure of eating salty foods, as hypothesized by Rose, then the individual ethical calculus would no longer be negative. Second, this discussion does not apply to behavior change that involves easily extinguishable preferences or habits that are easily adapted, such as seatbelt-wearing. On the other hand, it clearly applies to preferences that are rooted in evolutionary selection and reinforced by potent activation of the brain’s reward circuitry, such as an affinity for salty or fatty foods [20]. Third, implicit cost transfers need to be considered in the harm to benefit calculus (such as costs incurred to society by spending for salt-related health care costs). Fourth, this discussion assumes that health and wellbeing effects on different individuals do not have important communicable components, whether direct (a communicable pathogen) or indirect (an influencing behavior). Vaccines for COVID, influenza, and other highly communicable pathogens offer stark illustrations of how communicability may render population effects more ethically important than individual effects. Fifth, it is worth noting that an individual’s ethical calculus may be very sensitive to the stringency with which mandates pursue a health goal. For example, moderate reduction of salt in packaged foods, as has been accomplished in the U.K. and several other countries [24], may be ethically favorable because the gain in wellbeing from health benefits exceeds the mild loss in wellbeing. In contrast, stringent reduction of salt to less than 2 g per day may impair wellbeing sufficiently to outweigh health benefits for many people. Sixth, it is difficult to disambiguate empirically derived estimates of inequality-aversion (disliking the ethical consequences of unequal distributions) from risk-aversion (disliking the uncertainty regarding an individual’s position in a distribution as it becomes more unequal). Nonetheless, spillover from risk-aversion into inequality-aversion does not diminish the ethical relevance of that aversion. Finally, individual preferences may change, and therefore some scholars argue that any calculus that depends on the stability of preferences is inherently flawed. However, preferences rooted in evolution and reinforced by mesolimbic reward system exhibit great stability. While virtually all countries worldwide have attempted to reduce the salt intake of their populations, only 12 have documented subsequent reductions in daily salt intake. Among these 12 countries, the largest proportional reduction (China, 28%) was only sufficient to reduce salt intake to 12 g per day, and populations in all 12 countries continued to consume between 7 and 15 g per day, far above recommended levels [24]. In another example of the stability of reward-reinforced behaviors, the long-term success of non-procedural weight loss interventions is strikingly low (pooled estimate, 2.4 kg weight loss) [25].

## Conclusion

Healthful behavior change policies can be unethical. A policy may be unethical if its unfavorable non-health consequences outweigh its favorable health consequences sufficiently to compromise wellbeing for a substantial segment of the population, if the healthful behavior will be disproportionately adopted by those already healthy, or if the health benefit is offset by unintended consequences. Public health decision-makers should employ more structured, explicit, and comprehensive criteria when considering the ethical consequences of policies.
